# Psychiatric inpatient beds for youths in China: data from a nation-wide survey

**DOI:** 10.1186/s12888-020-02802-x

**Published:** 2020-08-06

**Authors:** Feng Geng, Feng Jiang, Jeffrey Rakofsky, Tingfang Liu, Yuanli Liu, Huanzhong Liu, Yi-lang Tang

**Affiliations:** 1 Department of Psychiatry, Chaohu Hospital of Anhui Medical University, No.64 Chaohu North Road, Hefei, 238000 Chaohu District China; 2 Hefei Fourth People’s Hospital, No.316 Huangshan Road, Hefei, 230022 China; 3 School of Public Health, Chinese Academy of Medical Sciences and Peking Union Medical College, No.3 Dong Dan San Tiao, Beijing, 100730 Dongcheng District China; 4 Research Center for Public Health, Tsinghua University, Beijing, 100730 Haidian District China; 5 Department of Psychiatry and Behavioral Sciences, Emory University, Atlanta, GA 30329 USA; 6 Institute for Hospital Management of Tsinghua University, Beijing, 100730 Haidian District China; 7 Mental Health Service Line, Atlanta VA Medical Center, Decatur, GA 30033 USA

**Keywords:** Child, Adolescent, Psychiatric, Hospitalization, China

## Abstract

**Background:**

The development of child psychiatric services in China has been slow and very limited resources have been allocated to support its growth. This study set out to investigate the child and adolescent inpatient psychiatric resources currently available in top-tier psychiatric hospitals in China and the characteristics of youth patients hospitalized on an adult unit.

**Methods:**

As part of an official national survey, 29 provincial tertiary psychiatric hospitals in China were selected. Data from 1975 inpatients discharged from these hospitals from March 19 to 31, 2019 were retrieved and analyzed.

**Results:**

The mean number of youth psychiatric beds was 27.7 ± 22.9 in these hospitals and 6/29 hospitals had no youth beds. There were significantly more youth beds in developed regions than in less developed regions (*P* < 0.05). Most of the discharged youth patients were teenagers with severe mental illnesses, including schizophrenia, depressive disorder and bipolar disorder. 7.5% (149) of the 1975 discharged patients were children or adolescents, however youth beds only accounted for 3.2% (804/25,136) of all psychiatric beds. 45.6% (68) of youth patients were discharged from adult psychiatric units.

**Conclusion:**

Our findings highlight the lack of adequate youth psychiatric inpatient services for children and adolescents living in China, especially in less developed regions. There is an urgent need to build more child and adolescent psychiatric units in provinces where there are none, and to increase the number of beds within the units that exist presently.

## Background

Mental disorders are common in children and adolescents. One recent meta-analysis of 41 studies in 27 countries showed the worldwide-pooled prevalence of mental disorders was 13.4% in children and adolescents [1]. China has a population of more than 1.3 billion, of which a quarter of a billion are children and adolescents between the ages of 5 and 19 [2]. Studies have shown that mental disorders in this age group are common in China, although no national data are currently available. For example, some regional surveys showed the prevalence of mental disorders in children and adolescents was approximately 20% [3], similar to that of many developed countries. Two studies in different regions in China found that the time-point (current) prevalence rates of major depressive disorder in 6 to 16 year-olds were 0.61 and 1.2% [4, 5]. Other studies showed the suicide attempt prevalence in adolescents was around 3% [6, 7]. Studies also showed psychotic disorders including schizophrenia were the most frequent diagnosis in youth inpatients [8, 9].

Most child and adolescent patients (youth patients, hereafter) with mental disorder can be and should be managed as outpatients in the community, to maximize the involvement of their families and other resources [10, 11]. However, for some severe cases or for those who are in acute crisis, psychiatric hospitalization remains a necessary and effective treatment option, although the duration has shortened significantly compared with what it was in the past [12–14].

Due to their unique developmental features, the consensus is that youth patients requiring inpatient care for mental health problems should be managed in age-appropriate facilities [15, 16]. In its policy statement, the American Academy of Child and Adolescent Psychiatry states clearly that “Unless there are compelling clinical reasons to the contrary, or serious limitations in availability, children and adolescents younger than 14 years of age should be admitted only to programs that are designed for children and adolescents and physically distinct from programs for adult psychiatric patients.” [17] Staff on adult wards often lack necessary training and confidence to care for youth inpatients [18]. Thus, psychiatric facilities should have appropriate resources for the treatment of youth inpatients, including a child and adolescent psychiatric ward and a sufficient number of beds reserved for this patient demographic.

Although child psychiatry has always been an important subspecialty of psychiatry in China, the overall development of the field in China has been slow as demonstrated by a scarcity of trained professionals, limited availability of treatment facilities, and a lack of funding and policy support [3, 19]. One recent survey showed that by the end of 2015 the psychiatric beds for children only accounted for 0.89% of total psychiatric beds in Mainland China, and only 175 out of 2936 (5.96%) psychiatric facilities in China had a child psychiatric ward [20].

When there are no age-appropriate facilities available or accessible for child and adolescents, some of these patients may have to be hospitalized on an adult unit. However, there are negative effects of mixing youth inpatients with adults. One study showed that over a quarter of adolescents engaged in criminal activity after they were admitted to an adult inpatient unit in Ontario, Canada [21]. Another report, based on the data from England and Wales, found that more than a third of youth psychiatric patients were admitted to general psychiatric wards and/or pediatric wards, and a review of those cases deemed more than half of these admissions as “inappropriate”. Additionally, the authors believed that these young patients may feel unsafe when mixed with adult inpatients, fearing assaults by the older patients [22].

In this study, using data from a nation-wide survey of top-tier psychiatric hospitals, we analyzed the percentage of child and adolescent beds in different hospitals and different regions, and we also compared the percentage of youth beds to the percentage of discharged youth patients to demonstrate the need and availability of inpatient services throughout the country. The basic clinical features of youth patients hospitalized in adult units were also analyzed.

## Methods

This study was a part of a large research project, the National Survey for the Evaluation of Psychiatric Hospital Performance [23]. We selected one representative provincial psychiatric hospital under the jurisdiction of the Ministry of Health (now incorporated into the new National Health Commission) in each province. We did not include Gansu and Tibet because there were no psychiatric tertiary hospitals at the time of survey. We also did not include hospitals within the jurisdiction of the Ministry of Public Security (Forensic psychiatric hospitals) and the Ministry of Social Welfare (Safety net hospitals) as their patient populations are different and they often follow different guidelines about staffing and resource allocations. In total, 29 psychiatric hospitals from 29 provinces and autonomous regions in Mainland China were selected and the basic data from each hospital including the total number of beds and the number of child and adolescent beds were collected and verified through government databases.

Information about discharged psychiatric patients between March 19 to 31, 2019 were retrieved, including demographic data and clinical features. The discharge diagnosis was based on the International Classification of Diseases and Related Health Problems (10th revision, ICD-10).

Summary statistics were used to describe the data. Comparisons of the number of child and adolescent beds and the percentage of youth beds in various areas were calculated using the one-way ANOVA test, while T-tests were used for the comparisons of beds and percentage of beds between developed regions and less developed regions. A Chi-square test was used to compare the ratios. The SPSS version 22.0 software (IBM Corp, Armonk, NY) was used to perform the basic statistical analyses.

All the tests were two-sided and statistical significance was defined as *P* < 0.05.

## Results

### Demographics and clinical characteristics of discharged youth patients

The mean age of the discharged youth patients was 15.3 ± 1.9 years old, with no significant differences between boys and girls (15.3 ± 1.9 vs. 15.3 ± 1.9). The majority, 58.4% of the youth patients, were girls. The youngest age of those patients was six years and most of the discharged youth patients were teenagers (13 to 17 years old, 93.3%). The three most common discharge diagnoses were schizophrenia (30.2%), depressive disorder (22.8%) and bipolar disorder (19.5%, all three accounted for 72.5%) (ICD-10).

### Numbers of youth beds and ratio of youth beds in tertiary psychiatric hospitals

In this survey, the number of youth beds varied greatly across different hospitals. Six of the 29 provincial hospitals did not have a single youth psychiatric bed (youth unit). These included Hainan, Jilin, Jiangxi, Ningxia, Chongqing and Qinghai provinces. There were 74 youth psychiatric beds in Anhui province, followed by Henan (73 beds), Hubei (65 beds), Liaoning (58 beds) and Beijing (57 beds). The average number of youth beds was 27.7 ± 22.9, with a median of 30. Table 1 shows the proportion of youth psychiatric beds out of the total number of psychiatric beds in each hospital, ranging from 0 to 7% (mean = 0.03 ± 0.02, median = 0.03).

**Table 1:** Number of discharged youth patients/all discharged patients and youth beds/total beds among different tertiary hospitals and regions

	**Number of youth patients**	**Total number of patients**	**Proportion of youth/total patients (%)**	**Number of youth beds**	**Number of total beds**	**Proportion of youth/total beds (%)**	***x***^**2**^	***P***
**North China (*N*** **= 5)**	**27**	**367**	**7.36**	**149**	**4075**	**3.66**	**12.116**	**< 0.001**
Beijing	**13**	**104**	**12.5**	**57**	**800**	**7.13**		
Tianjin	**6**	**66**	**9.09**	**32**	**1220**	**2.62**		
Shanxi	**0**	**72**	**0**	**10**	**625**	**1.6**		
Inner Mongolia	**1**	**55**	**1.82**	**35**	**820**	**4.27**		
Hebei	**7**	**70**	**10**	**15**	**610**	**2.46**		
**Northeast China (*N*** **= 3)**	**0**	**184**	**0**	**63**	**2391**	**2.63**	**–**	**–**
Liaoning	**0**	**52**	**0**	**58**	**1532**	**3.79**		
Jilin	**0**	**82**	**0**	**0**	**429**	**0**		
Heilongjiang	**0**	**50**	**0**	**5**	**430**	**1.16**		
**East China (*N*** **= 7)**	**51**	**516**	**9.88**	**216**	**6945**	**3.11**	**63.866**	**< 0.001**
Shanghai	**9**	**104**	**8.65**	**25**	**2141**	**1.17**		
Jiangsu	**12**	**110**	**10.91**	**30**	**500**	**6.0**		
Zhejiang	**7**	**50**	**14.0**	**32**	**1054**	**3.04**		
Anhui	**2**	**50**	**4.0**	**74**	**1280**	**5.78**		
Fujian	**3**	**60**	**5.0**	**20**	**570**	**3.51**		
Jiangxi	**5**	**62**	**8.06**	**0**	**660**	**0**		
Shandong	**13**	**80**	**16.25**	**35**	**740**	**4.73**		
**Central South China (*N*** **= 6)**	**53**	**525**	**10.10**	**239**	**6111**	**3.91**	**43.955**	**< 0.001**
Hubei	**12**	**121**	**9.92**	**65**	**950**	**6.84**		
Hunan	**9**	**71**	**12.68**	**26**	**562**	**4.63**		
Guangdong	**14**	**100**	**14.0**	**40**	**1611**	**2.48**		
Hainan	**1**	**51**	**1.96**	**0**	**850**	**0**		
Henan	**7**	**100**	**7.0**	**73**	**1500**	**4.87**		
Guangxi	**10**	**82**	**12.20**	**35**	**638**	**5.49**		
**Southwest China (*N*** **= 4)**	**12**	**228**	**5.26**	**57**	**3314**	**1.72**	**14.021**	**< 0.001**
Sichuan	**2**	**62**	**3.23**	**12**	**1257**	**0.95**		
Yunnan	**3**	**51**	**5.88**	**40**	**982**	**4.07**		
Guizhou	**4**	**55**	**7.27**	**5**	**535**	**0.93**		
Chongqing	**3**	**60**	**5.0**	**0**	**540**	**0**		
**Northwest China (*N*** **= 4)**	**6**	**155**	**3.87**	**80**	**2300**	**3.48**	**0.066**	**0.797**
Shaanxi	**2**	**58**	**3.45**	**45**	**700**	**6.43**		
Qinghai	**1**	**20**	**5.0**	**0**	**300**	**0**		
Ningxia	**1**	**21**	**4.76**	**0**	**326**	**0**		
Xinjiang	**2**	**56**	**3.57**	**35**	**974**	**3.59**		
**Developed regions**^**a**^***(N = 13)***	**110**	**1082**	**10.17**	**504**	**13,575**	**3.71**	**103.991**	**< 0.001**
**Less developed regions**^**b**^***(N = 16)***	**39**	**893**	**4.37**	**300**	**11,561**	**2.59**	**9.834**	**0.002**
**Total**	**149**	**1975**	**7.54**	**804**	**25,136**	**3.20**	**101.96**	**< 0.001**

**a.** Developed regions: Guangdong, Jiangsu, Shandong, Zhejiang, Henan, Sichuan, Hubei, Hunan, Hebei, Fujian, Shanghai, Beijing and Anhui;

**b.** Less developed regions: Liaoning, Shaanxi, Jiangxi, Chongqing, Guangxi, Tianjin, Yunnan, Inner Mongolia, Shanxi, Heilongjiang, Jilin, Guizhou, Xinjiang, Hainan, Ningxia, Qinghai

### Regional differences in youth beds and bed ratio

We divided 29 provinces into 6 regions based on the official geographic regional classification: North, Northeast, East, Central South, Southwest and Northwest China. The mean number of youth beds in six geographic regions ranged from 14.3 to 39.8 across six regions of China, while the mean percentage of youth beds ranged from 1.72 to 3.91%. No significant regional differences were found.

China is a geographically vast and socioeconomically uneven country. We used the official GDP data of 2018 to group the 29 hospitals into developed (*N* = 13 hospitals) and less developed (*N* = 16) regions. We found the hospitals in developed regions had significantly more child and adolescent beds (38.8 ± 21.5) and a higher percentage of youth beds (4.1%) than those in less developed regions (18.8 ± 20.4, 2.1%) (t = 2.568, df = 27, *P* = 0.016 and t = 2.511, df = 27, *P* = 0.018 respectively).

### The percentage of youth beds and the percentage of discharged youth patients

During the study period, 1975 patients were discharged from the participating hospitals, and 149 (7.5%) were youth patients (< 18 years old) and 1826 were adult (≥18 years old). In the meantime, the proportion of youth beds in these hospitals was only 3.2% (804/25,136). A significant difference was found between the percentage of discharged youth patients and the percentage of youth beds (*p* < 0.001). Further, significant results were obtained when making these same comparisons within the different regions, excluding Northwest China. Ratios were not compared in Northeast China because there were no youth patients discharged during the study period.

Among 149 discharged youth patients, 68 (45.6%) were discharged from adult psychiatric units. Since six hospitals had no youth beds, we excluded data from those six hospitals, and we found 43.1% (57/138) of the discharged youth patients had been discharged from an adult unit. 2.7% (42/1541) of discharged adult patients were discharged from a child and adolescent unit. These adult patients ranged from 18 to 80 years old with a mean of 38.83 ± 15.90, and only two of them were exactly 18 years old.

## Discussion

This is the first study focusing on youth psychiatric beds in China using data collected from a nation-wide survey. A few important findings must be highlighted. First, we confirmed that overall there is a scarcity of child and adolescent psychiatric beds in China and in six provincial regions, there are none at all. As expected, more of these beds were found in hospitals located in socioeconomically developed regions. Additionally, based on the data from discharged patients, more than two fifths of child and adolescent patients had been hospitalized on adult inpatient units.

A few limitations also must be acknowledged: First, due to the nature of the design (cross-sectional) and a lack of important contextual data, we were unable to determine the actual number of youth patients who were turned away because there were no available beds and the unmet needs for certain areas. Second, all the participating hospitals were tertiary hospitals and they often are the largest and have the most healthcare resources. While this makes our findings less generalizable to other areas, it is probable that the situation is worse in less developed or rural areas. Third, although we estimated the percentage of youth patients who had been admitted to adult units, we have no individual-level data on the patients; therefore, we were unable to analyze the differences between patients who were admitted to a child and adolescent unit versus those who were admitted to an adult unit. Fourth, discharge data were used as a proxy for inpatient capacity. However, the number of patients discharged in a two-week period may not be an accurate reflection of inpatient capacity, especially if some patients were hospitalized for longer than two weeks.

Most of the youth patients were between 13 to 17 years old and the three most common discharge diagnoses were schizophrenia (30.2%), depressive disorder (22.8%) and bipolar disorder (19.5%). As data were extracted from discharge face sheets, the exact reasons for hospitalization were not captured. It is reasonable to assume that some of the common reasons were suicidality or self-harm behavior, acute exacerbation of psychosis, and risk of harm to others. Two recent studies showed that the rate of suicide attempts was around 3% in adolescents, and most first suicide attempts occurred around age 12 or 13 [6, 7].

In China, most healthcare resources (including mental healthcare) are located in public hospitals. As a result, the participating psychiatric hospitals likely represent the healthcare resources in each province. From our survey, only half of these psychiatric hospitals had more than 30 youth beds, while more than half of those provinces have more than seven million children and adolescents between 5 and 19 years olds [2]. Six hospitals have no youth units, which means many youth patients and their caregivers have limited options and the children may have to be hospitalized on a unit mixed with adults. Of note, although there is a youth psychiatric unit in some provinces (often located in the capital), it is likely to be the only one in the entire province, so accessibility is extremely limited. Although determining the appropriate number of inpatient beds for children and adolescents is hard and often challenging [24], the current situation is clearly unacceptable, and it is imperative to establish child and adolescent psychiatric units in all provinces.

In China, public inpatient psychiatric services are the main healthcare resource for patients with severe psychiatric illness [25]. Despite greater recognition of the importance of mental health promotion and prevention for children and adolescents in China [26], there still is an enormous discrepancy between needs and inpatient service availability. This so called “access gap” happens more frequently in low-and middle-income countries than high-income countries [27]. One study showed less than 1% of beds in inpatient facilities are available to children and adolescents in these countries [28]. This significant gap was also shown in our study, although our data were limited to the top-tier psychiatric hospitals.

The percentage of discharged youth patients in our study was 7.5%, which was similar to the results from a U.S. study that reported that children and adolescents represent approximately 7% of the total mental health inpatient population [29]. Meanwhile, the proportion of youth beds in our study was only 3.2%. This is an indication that resource allocation in China for child and adolescent patients is not proportional to the need. Despite the discrepancy in these percentages, there may actually be enough beds for child and adolescent patients throughout China, but unfortunately, they are not distributed proportionately throughout the provinces, leaving psychiatrists in some of these regions with no choice but to admit their child patients onto adult units.

Several concerns have been raised about this practice: First, youth patients often do not feel comfortable among adults with mental disorders [30, 31]. Second, an adult psychiatric unit often has different physical features and designs that may not be appropriate for younger patients [32, 33]. Third, staff on adult wards often lack necessary experience or training and they do not feel confident working with young patients [18]. At least one study showed that some young patients reported feeling dissatisfied during their time on adult psychiatric wards [34]. The general consensus is that adult wards are inappropriate for child and adolescent patients with mental illness [16].

## Conclusion

This study provides important data about the current number and distribution of child and adolescent beds in top-tier psychiatric hospitals across China. The overall situation is dire and requires the immediate attention of policymakers and administrators, especially in those provinces where there are currently no child and adolescent psychiatric wards. Establishing these inpatient units where there are none, and increasing the number of youth beds on units that already exist should be a priority as children and adolescent patients will receive the best care when treated in age-appropriate facilities.

## Data Availability

The datasets used and/or analyzed during the current study are available from the corresponding author on reasonable request.
